# Use of electronic personal health record systems to encourage HIV screening: an exploratory study of patient and provider perspectives

**DOI:** 10.1186/1756-0500-4-295

**Published:** 2011-08-15

**Authors:** D Keith McInnes, Jeffrey L Solomon, Barbara G Bokhour, Steven M Asch, David Ross, Kim M Nazi, Allen L Gifford

**Affiliations:** 1 Center for Health Quality, Outcomes & Economic Research, ENRM VA Medical Center, Bedford, MA, USA; 2 Boston University School of Public Health, Boston, MA, USA; 3 VA Palo Alto Health Care System, Palo Alto, CA, USA; 4 Division of General Medical Disciplines, Stanford School of Medicine, Stanford, CA, USA; 5 VA Public Health Strategic Health Care Group, Washington, DC, USA; 6 VA Veterans and Consumers Health Informatics Office, Washington, DC, USA; 7 Boston University School of Medicine, Boston, MA, USA

## Abstract

**Background:**

When detected, HIV can be effectively treated with antiretroviral therapy. Nevertheless in the U.S. approximately 25% of those who are HIV-infected do not know it. Much remains unknown about how to increase HIV testing rates. New Internet outreach methods have the potential to increase disease awareness and screening among patients, especially as electronic personal health records (PHRs) become more widely available. In the US Department of Veterans' Affairs medical care system, 900,000 veterans have indicated an interest in receiving electronic health-related communications through the PHR. Therefore we sought to evaluate the optimal circumstances and conditions for outreach about HIV screening. In an exploratory, qualitative research study we examined patient and provider perceptions of Internet-based outreach to increase HIV screening among veterans who use the Veterans Health Administration (VHA) health care system.

**Findings:**

We conducted two rounds of focus groups with veterans and healthcare providers at VHA medical centers. The study's first phase elicited general perceptions of an electronic outreach program to increase screening for HIV, diabetes, and high cholesterol. Using phase 1 results, outreach message texts were drafted and then presented to participants in the second phase. Analysis followed modified grounded theory.

Patients and providers indicated that electronic outreach through a PHR would provide useful information and would motivate patients to be screened for HIV. Patients believed that electronic information would be more convenient and understandable than information provided verbally. Patients saw little difference between messages about HIV versus about diabetes and cholesterol. Providers, however, felt patients would disapprove of HIV-related messages due to stigma. Providers expected increased workload from the electronic outreach, and thus suggested adding primary care resources and devising methods to smooth the flow of patients getting screened. When provided a choice between unsecured emails versus PHRs as the delivery mechanism for disease screening messages, both patients and providers preferred PHRs.

**Conclusions:**

There is considerable potential to use PHR systems for electronic outreach and social marketing to communicate to patients about, and increase rates of, disease screening, including for HIV. Planning for direct-to-patient communications through PHRs should include providers and address provider reservations, especially about workload increases.

## Background

Arguments for expanding HIV screening are compelling. When detected, HIV can be effectively treated with antiretroviral therapy (ART), which improves patient survival, helps prevent HIV transmission, and is cost effective [1-3]. Nevertheless in the U.S. approximately 25% of those who are HIV-infected do not know it [4,5]. Much remains unknown about how to increase HIV testing rates in the US.

Direct outreach to patients via the Internet is a potentially efficient means of educating patients about the importance of HIV screening. Patient electronic personal health record (PHR) systems may be a useful vehicle for such outreach [6-8]. Little is known, however, about how patients and healthcare providers would perceive use of the PHR to disseminate disease screening messages, or whether such messages would increase HIV testing, e.g. by increasing patient knowledge [9], self-efficacy [10], and activation [11]. Additionally this type of outreach could raise patient concerns about privacy of information on the Internet, especially for stigmatized conditions like HIV. Providers may have concerns that workload will increase, or that direct-to-patient outreach circumvents provider authority. While electronic outreach for health purposes is not new, it has largely been evaluated in the context of randomized trials of specific interventions [12-14], or newsletters for which consumers pro-actively register [15]. Little is known about how providers and patients within a large health care organization would perceive large-scale, unsolicited, outreach via an electronic personal health record system to encourage HIV screening.

As the largest provider of HIV care in the U.S., the Department of Veterans Affairs (VA) is well suited for evaluating different methods for increasing HIV testing. The VA already devotes considerable effort to increasing HIV screening rates [16], including clinical reminders in the electronic medical record, provider performance profiling, and reducing paperwork barriers to testing [17-19]. Still, testing rates are sub-optimal, with an estimated 20% to 50% of VA patients with documented risk factors for HIV infection having been tested [20-22]. The VA's electronic PHR, My Health*e*Vet, contains email addresses of nearly 1,000,000 veterans, most of whom (87%) report using VA health care [23]. Thus the VA is an appropriate system for implementing and evaluating large scale electronic outreach for HIV screening. The VA PHR was (and is) evolving rapidly, with new versions released approximately every 6 months (current version is 11.2). In addition, at the time of this study the VA was preparing to adopt new Centers for Disease Control and Prevention (CDC) recommendations for routine, instead of risk-based, HIV testing. Among other things this involved the elimination of the requirement to obtain written patient consent prior to testing. In the context of this rapidly changing landscape we selected methods which would quickly provide VA policy makers with preliminary patient and provider perceptions of the use of the PHR to encourage more HIV screening.

We explored patient and provider attitudes toward an electronic outreach program for HIV screening, based on a PHR platform. We conducted focus groups with patients and providers about HIV testing. We also discussed diabetes and cholesterol screening with participants to assess whether attitudes toward outreach to increase screening depended on the health condition. We explored the acceptability of messages embedded directly in personal emails versus messages posted on the PHR website. Our focus group guides were informed by the Information-Motivation-Behavioral Skills (IMB) model which has guided health promotion and chronic disease management, including HIV [24,25]. We used the model to guide broad categories of questions to include in the focus groups. Qualitative methods were used because, with such new areas of research, it is important to identify salient patient and provider perceptions and themes prior to embarking on larger scale quantitative research [26-28].

## Methods

### Overview

**Figure 1:**
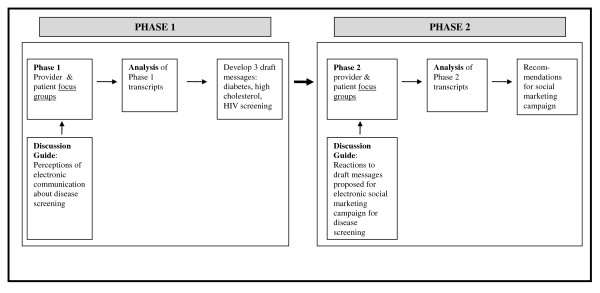
**Study components and flow**.

Four focus groups were conducted between September 2008 and March 2009, in two phases (Figure 1). The first phase explored HIV screening (and other disease screening) in general terms and sought participant suggestions about the content and framing of the electronic outreach messages. Results from this phase guided the investigators in drafting the content of messages. In the second phase, we presented participants the draft text of HIV, diabetes, and cholesterol screening messages in order to explore patient and provider perceptions of realistic content. It was important to compare perceptions of other chronic health conditions to HIV in order to assess whether HIV-related stigma would adversely affect acceptance of HIV screening messages. The message text was based on VA and Centers for Disease Control and Prevention (CDC) screening guidelines, as well as findings from the phase 1 focus groups. The Institutional Review Board of the Edith Nourse Rogers Memorial VA Medical Center, Bedford, MA approved the study including all recruitment methods. Study subjects completed written informed consent prior to participating.

### Participants and Setting

A total of 12 patients (6 in each focus group) and 15 providers (6 in one focus group, 9 in the other) participated. Patients were recruited from a Boston area VA medical center. We posted recruitment fliers on walls and a "crawler" message on the televisions in the medical center waiting areas. We also approached veterans at new-patient orientation sessions and in the veterans computer center (KM and LM). Patients received $20 for their participation.

Primary care providers were recruited from another urban New England VA medical center. The invitation to providers was extended by a research team member (KM) who described the study at a primary care staff meeting. Providers were not compensated for participating.

### Procedures

**Figure 2:**
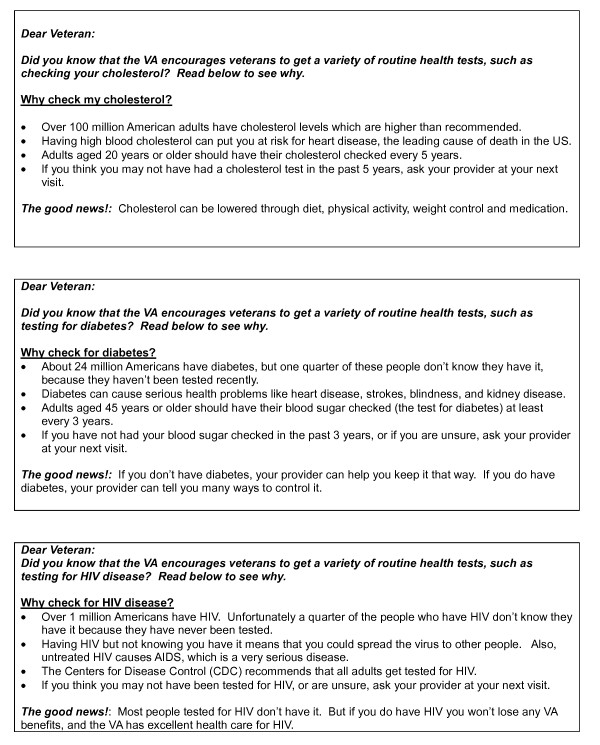
**Text of electronic messages shown to patients and providers in focus groups**.

There were two facilitators (KM and JS) for each focus group. Following the IMB model, we developed focus group guides to explore whether the concept of electronic disease screening messages, and message content, were perceived as providing valuable information. Secondly questions assessed how likely the information was to motivate patients to seriously consider being tested, and how likely they would be to take action, i.e. ask their provider for a test (behavioral skills). More specifically, the phase 1 patient focus group guide covered experience with disease screening; sources of information about disease screening; experience with Internet and My Health*e*Vet; and, attitudes toward the proposed VA electronic outreach program to increase disease screening rates. The phase 2 patient guide elicited reactions to draft texts of messages for HIV, cholesterol, and diabetes screening (Figure 2) that might be part of the VA disease screening outreach program. Patient focus groups lasted two hours.

The phase 1 provider focus group guide elicited discussion of how providers decide to screen individual patients for diseases; provider views of patient requests for disease screening; provider perceptions of the proposed electronic outreach program; and their assessments of how patients would react to such a program. The phase 2 guide asked providers for their reactions to the same draft messages (for HIV, cholesterol, diabetes) that were shown to patients (Figure 2). They were asked how they anticipated their patients would respond, what questions their patients might ask, and what they thought patient reaction would be. Provider focus groups lasted one hour.

While the research was conducted in two phases, with separate focus group guides for each phase, the results are presented by themes, rather than phase. This is because there was considerable overlap between the focus group guides from the first and second phases. Hence the themes we uncovered emerged from all four focus groups. We have indicated after each quote which phase it came from, e.g. "Patient FG1" refers to first phase patient focus group. Because the focus group guides are lengthy (they make extensive use of probes and prompts that the focus group facilitator can use at his or her discretion) they are not included here, however they are provided for interested readers in Additional File 1: Focus group guides used for patients and providers. All focus groups were audio-taped and transcribed.

### Analysis

We used an iterative process to guide the analysis and interpret data, based on grounded theory methods [29]. Immediately following each focus group the facilitators discussed their impressions of significant points that emerged from the focus group. In addition, within a week the facilitators briefed the whole research team, summarizing the focus group content, and eliciting comments about emerging themes. Audio-recordings were transcribed verbatim by a professional transcription firm. Focus group facilitators (KM and JS) verified the transcripts and analyzed them by open coding, i.e. identifying key concepts emerging from the language used by participants, and assigning codes (descriptive phrases) to segments of text. NVivo qualitative analysis software (QSR, Melbourne, Australia) was used to facilitate data coding and sorting. Coded text segments were reviewed by three investigators (KM, JS, and BB) to categorize codes into distinct themes. Where similar themes were identified in patient and provider transcripts, we examined similarities and differences in patient and provider perspectives. In a final phase, after developing preliminary interpretations, we searched through the data for alternative interpretations and rival conclusions.

## Results

### Participant Characteristics

Patients ranged in age from 48 to 71 years of age. Most were white and male. Two-thirds were Internet users (used email and/or the Internet). All had some college education. The 5 physicians and 7 primary-care nurse practitioners participating ranged in age from 46-60 years (see Table 1).

**Table 1:** Characteristics of focus group participants

	Patient Focus Groups	Provider Focus Groups
Number	6 in group 1	6 in group 1
	6 in group 2	9 in group 2*
Gender	2 female; 10 male	8 female; 4 male
Race/Ethnicity	9 white	[Not collected]
	1 African American	
	1 Hispanic	
	1 Pacific Islander/Hawaiian	
Education/Qualifications	6 some college or college degree	7 nurse practitioners
	3 some graduate or graduate degree	5 medical doctors
	3 not provided	
		
Age	48 to 71 years	46 to 60 years

* There were 12 unique providers because 3 providers participated in both focus groups.

### Overview of findings

Patients and providers perceived important informational and educational benefits of the proposed electronic outreach. Several providers expressed substantial privacy concerns related to the social stigma associated with HIV. Patients, for the most part, did not perceive HIV messages to be inherently more sensitive than messages about diabetes and cholesterol. Providers anticipated increased workload and made recommendations for message content in order to minimize disruption to primary care practice.

### Perceived benefits for patients of screening messages in general

#### The more information the better

Patients and providers perceived that electronic disease screening outreach would improve patient access to useful health information, with important educational value. For providers there was a perception that it would reinforce messages they give to patients. Patients seemed interested in more information, and saw this outreach as a potentially good way to achieve this goal. Here, a patient expresses his view that too many people take their bodies and their health for granted, and that the messages proposed could help combat this complacency.

"I think all this information would be great. Because I think how else are we going to know what to do with the only true asset we own [which] is our body. And some people spend more time getting the oil changed in their car than they do worrying about what's going on in [their bodies]." *(Patient FG2)*

Providers realized that their repeated recommendations to patients to be screened lose effectiveness. Using a new medium, i.e. the Internet, could be a useful adjunct to what providers are trying to communicate to their patients.

"I think for established patients, this is reinforcing education. The last sentence [of the draft text shown to providers], 'Cholesterol can be lowered,' they're hearing that all the time from us. And now they're reading it, so [it's] another teaching tool." *(Provider FG2)*

#### Information using lay language and available when patients are ready for it

Patients could imagine scenarios in which disease screening information provided electronically would be better than verbal information from their doctor. The patient below knows there are times when other factors, in this instance substance use, interfere with his ability to absorb important messages from his doctor.

"Let's say I went in from detox. [My doctor] might be saying all this stuff to me but I might be in a situation where I'm like, 'I ain't listening to all this stuff at this point now.' When my head starts to clear out [I might think] 'Okay. What did this doctor say?'" (*Patient FG2*)

An electronic message gives the patient another opportunity to receive the information, and the choice of when and how many times to read it. These messages can be carefully worded to accommodate low literacy levels, as expressed by this patient,

"If you put [the web information] in layman's terms pretty much explaining LDL or HDL...and how you get it, [that's better than having] $20 dollar words in there." (*Patient FG2)*

#### Messages can motivate patients

Patients felt that electronic outreach would motivate them to be proactive about their health. Most felt the electronic messages would remind them to be screened, or at least contemplate getting screened. Here a patient finds the idea of an email about HIV screening to be non-threatening, and potentially motivating.

"They're not telling you [you have to be tested for HIV]. They're putting it in your mind saying... "Have you ever thought about getting HIV testing?" It's non-offensive. You're not prying. But it gets you thinking. Something like that might work." (*Patient FG1*)

Below, two patients, discussing diabetes screening, conclude that outreach messages would be valuable, despite their different perceived risk for the condition. The first realizes that a common "if it's not broken, don't fix it" attitude, may prevent people from thinking about getting preventive testing.

"As far as [an email] prompting you to go and get [a test] done, yeah there's probably people that aren't even aware that they should have them. Up until five years ago, I never thought about getting my blood sugar checked. What do I care? It's not bothering me any." *(Patient FG2)*

The second patient has a family history of diabetes that he/she might inadvertently ignore. Periodic reminders can be the extra motivation to take action and get tested.

"My father has diabetes. My mother is borderline diabetes [sic]. I've been checked periodically through the years and I don't seem to be having it...It might slip my mind where I'm not thinking I'm going to get it...and then all of a sudden I see [the electronic message about diabetes screening] and I say, 'Maybe I ought to go and have it checked.' So it's kind of like a kick in the pants." (*Patient FG2*)

### HIV content: patient acceptance, provider wariness

Our focus group questions sought to contrast electronic outreach for non-sensitive conditions (i.e. diabetes and cholesterol) with HIV, a stigmatized condition. Few patients, however, made this distinction. Patients thought electronic messages about HIV were acceptable and useful, especially if they were clearly written as public health announcements for wide distribution. One patient likened HIV information delivered electronically through the PHR to posters about HIV testing found in many VA medical center waiting rooms; while another felt that because the material was for a generic patient audience it would not raise objections:

"I wouldn't mind [getting a message about HIV testing]; it's pasted all over the walls of the VA. I mean, I think the information is good." (*Patient FG2*)

"None of this is laden with any personal information on yourself or anything like that...I can't see any of this being upsetting to anybody." *(Patient FG2)*

A third patient, however, speaking about messages sent to personal email addresses, was worried about possible security breaches and the stigma of being associated with HIV. He suspected that once information entered his computer it would be difficult to erase, thus allowing later users to find such messages.

"I don't want 'You get tested for HIV' [in an email]... I've given away computers I've had to people who never had one... They can get into your mainframe, as you folks may know. They can find stuff that you left in there. I'm not taking that chance.... I'm very careful about what goes in my computer. I have a disk that I put everything on. I don't let it go on my mainframe. But some stuff goes in there. You think I want to take a chance and let HIV go in there? And they accidentally find it? Hell, no!" *(Patient FG1)*

This type of concern supports placing the disease screening messages on the PHR website, rather than delivering it directly into patient email inboxes. This sentiment is summarized by a patient in the first focus group (referring to the PHR by its VA name, "My Health*e*Vet"):

"I would like to see [a message in my personal email stating] "You have messages at My Health*e*Vet." That's all I want to see. Just tell me to go My Health*e*Vet website, log in and I get messages there. I'd rather see a message there than coming into [my personal email]." *(Patient FG1)*

Providers aired substantially more concerns about HIV messages than patients. Some providers felt that patients would be irresponsible with emails containing HIV-related content. The provider below, for example, described how patients easily find doctor email addresses, and could send their doctors inappropriate email. The provider expresses two issues: the risk that the patient becomes associated, in other people's minds, with a stigmatized condition, and the risk that providers get criticized from their employer for participating in inappropriate email use.

"I see a lot of problems with this, because there are going to be some [veterans] who aren't thinking about confidentiality. And they're going to be emailing their provider, which is 'my name-dot-VA-dot-gov'; And they're going to be saying 'Oh, I got this thing on HIV. I think I should be tested.' And it's going to be out there in the Internet world, floating around. And the VA is going to get dinged - or me - for 'Oh my God, why did this person email you about this?'" *(Provider FG2)*

This provider expresses the view, correct in some instances, that regular email messages are vulnerable because they "float around" in the Internet easily opened and read by other Internet users.

Another aspect of provider resistance toward HIV-related emails was that they could create suspicions among patients that the VA is withholding information from patients:

"And I think if you sent them an email, there are some people who might be walking in the next day, 'I got this email that told me to come in and be tested!...Why are you worried?...Why'd you send it to me? Did you send it to anybody else?'" (*Provider FG1*)

This view may reflect provider sensitivity to claims by veterans and active duty military that the US government releases too slowly important health-related information, especially for risks related to military service [30].

Finally, another provider's hesitation was that the HIV message was inappropriate because it was promoting a substantial deviation from the way providers recommended HIV testing. One provider remarked, "...this third [message] on HIV is like a bombshell," because it recommended routine HIV testing. Providers had described in the first focus group that they typically recommend HIV testing to their patients only if risk factors were present, i.e. intravenous drug use or men having sex with men. A consequence of these concerns seemed to be that providers preferred, if an outreach program were conducted, that content be posted on the PHR website, rather than transmitted via email. Patients and providers approved of a "tickler" email message to patients that would indicate there is new content on the PHR, with a hyperlink to the PHR website.

### Perceived provider burden

A prominent provider concern was that electronic outreach for disease screening would lead to unmanageable workload. They anticipated the outreach would result in a substantial increase in patient phone calls, time spent explaining and clarifying the outreach program, and additional appointments.

"If the VA is going to send out a newsletter [about disease screening],...especially if you're sending it electronically,...you're going to get this flood of phone calls the day it goes out, and probably the next week. And, if you're not prepared for that, you've got to have your telephone staff prepared. You have to have your primary care nursing staff prepared, your primary care provider staff. Because these things have this, like, volcano effect." (*Provider FG2*)

One provider suggested that the messages should contain preemptive language to discourage patients from immediately calling or visiting their provider:

"If you maybe send out [an electronic] newsletter [to patients that says] '... your provider will be asking you for A, B, C, D, E, F, G at your annual - highlighted, underlined, in bold, different color - visit', so [the patients realize] you don't need a PSA every time you come to the walk-in." (*Provider FG1*)

These providers did not reject the electronic outreach initiative, but have suggested that to be successful, it would be wise to make advance preparations with staff and to include education of patients that indicates this is not urgent and can be handled at annual - or other regularly scheduled visits. Other providers concurred, but also reflected a feeling that PCPs are being shouldered with increasing demands and performance measures, often without increases in resources:

"How can I do this? I want to be doing X, Y, and Z, and you're adding another element that I'm responsible for." *(Provider FG1)*

Patients, interestingly, did not indicate they would rush to contact their providers or make appointments to see their doctors as a result of electronic messages. In fact some patients believed that if the electronic communication had links to more information it might actually save doctors time:

"If you need more information...instead of having an hour conversation with the doctor and having the doctor teach you, you could actually go to a place on [the patient website for more information]." (*Patient FG2*)

## Discussion

As health care systems adopt new information technologies it is appropriate to consider their use for public health purposes, such as disease screening. This study takes a first, exploratory step in evaluating the acceptability of outreach via an electronic PHR system by soliciting patient and provider perceptions through focus groups. We found that perceptions were, on the whole, positive. Patients and providers acknowledged educational, informational, and motivational benefits of electronic messages. Providers especially, and patients to a much smaller degree, expressed privacy concerns about messages that contained HIV content. Those concerns could be mitigated by posting patient content on the PHR website, as opposed to embedding it in personal email messages. A bigger issue for providers, however, was that this kind of outreach could lead to unacceptable increases in workload. They suggested it could be mitigated by increased primary care resources and management of patient flow so that most additional disease screening could occur during annual visits or spread more evenly over time.

Patients indicated that electronic content afforded the ability to view information when and where convenient, at appropriate reading-levels, and with web-links to multiple sources of information. Using individual email addresses, however, carries the potential risk of creating suspicions among patients that they have been contacted based on specific clinical signs of HIV, or based on HIV risk stereotypes, e.g. homosexuality or intravenous drug use. HIV-related stigma also seemed to underlie provider worries that patients would unwittingly expose themselves to stigma if they sent their doctors emails about HIV testing. Accordingly, patients and providers favored an outreach approach that delivered content impersonally, i.e. posted on the healthcare system PHR website.

Our findings support the IMB model in that both patients and providers indicated that the electronic messages were perceived as providing important information, and that they would lead to patient action in terms of inquiries about, or actual increases in, testing. The findings also highlighted to us that the health belief model (HBM) [31] could be an important addition to the IMB model in helping to understand patient and provider responses to electronic messages about disease screening. This makes sense in that the health belief model often guides health-related social marketing campaigns [32-35] that rely on perceived susceptibility to disease to motivate people to take action. Patients with high perceived susceptibility may seek information, screening, and care on their own. Others patients, however, may have consciously or unconsciously suppressed the knowledge that their family history or risky health behaviors could make them vulnerable to certain health conditions. For such patients the electronic messages serve as external cues ("cues to action" in the terminology of HBM) motivating them to take action and get tested. While the current draft messages (Figure 2) incorporate concepts of information and motivation from the IMB model, future versions could have links to skill-building material - another important IMB component. For example the HIV message could link to material about how to have a conversation with a partner about using condoms, while the diabetes message could link to simple instructions for increasing daily physical activity.

It is noteworthy that the participating patients were middle aged and older adults, most of whom were not highly experienced computer and Internet users. Nevertheless nearly all recognized advantages that such technologies provide in distributing beneficial health information, a finding supported by Pew Research Center findings that older adults are increasing their presence online [36,37].

Provider concerns that electronic communications with patients may create unmanageable workloads have been documented previously [38]. Evidence suggests, however, that patient use of PHRs, secure messaging, and similar electronic communication tools do not overwhelm providers [39,40]. There is even evidence that electronic communication reduces in-person and telephone communication [6]. We found support for this, e.g. a patient stating that accessing information from a website could replace "an hour conversation with the doctor". The above notwithstanding, we do not dismiss provider concerns about increased workloads. Primary care providers face health system demands for better quality of care at lower costs, with resultant increased stress and loss of autonomy [41,42]. On the other hand solutions exist to even out demands on providers, for example by staggering electronic outreach messages based on patient birth dates or social security numbers.

The success of HIV screening campaigns may rest partly on patient perceptions that, in the event of a positive HIV test, they can gain access to compassionate providers and effective treatments, i.e. there is good linkage to care [43-45]. When HIV screening outreach is conducted by a health facility or system that has strong HIV care programs, it is likely the outreach will be more successful. In this regard, the VA would seem to be especially well suited to employ the kind of electronic outreach described in our study because it is a large, comprehensive health care system with specialized HIV clinics to care for veterans with HIV/AIDS. Currently approximately 23,000 veterans with HIV are in treatment in the VA [46].

This study was conducted 6 months before the VA formally adopted CDC guidelines which recommend routine HIV testing for all adults in care, regardless of risk factors [47]. This policy change eliminated written patient informed consent for HIV testing, in favor of verbal consent. Thus our findings represent patient and provider perceptions prior to implementation of the new HIV testing policy in the VA. Adherence to the CDC guidelines is far from universal even after the policy change in the VA and in other settings [48], suggesting the importance of continued outreach to patients to encourage HIV testing. In addition even in settings where the guidelines are closely adhered too, there will be patients who come for care infrequently and thus would benefit from this kind of outreach; the outreach messages might prompt them to make a visit or a phone call to discuss testing with their provider. In any case it will be important to evaluate whether patient and provider perceptions of HIV testing messages have changed in the VA and to extend the analysis to non VA sites.

As PHR systems continue to expand their capabilities, it is easy to imagine moving from occasional broad electronic outreach programs to more routine patient reminders that patients see when they open up their PHR. In the VA, for example, the PHR has recently implemented reminders for preventive care and procedures, such as diabetes care (foot and retinal exams), cancer screening, and immunizations [49]. It would not be difficult, technically, to add HIV screening to that list.

## Limitations

Our study was limited to 2 patient focus groups and 2 provider focus groups conducted in one region of the U.S. Thus our findings may not be generalizable to other regions and other healthcare systems. Only 2 female patients participated (1 in each group), also limiting generalizability. Our participants were middle-aged and older, and thus probably reacted differently to some issues than would participants in their 20s and 30s who have grown up with computers. Also participant responses might have differed had they been reacting to a "live" electronic outreach program rather than a proposed one. Our use of draft text, however, which participants reviewed in the focus groups, is likely to have created a sense of concreteness and immediacy.

## Conclusions

The growth in online information systems connecting healthcare organizations with their patients provides an excellent opportunity to conduct low cost and potentially high impact electronic outreach and social marketing. Our findings suggest that patients and providers endorse the use of PHRs for disease screening outreach, even for a stigmatized health condition such as HIV. For providers it is important that prior to initiating wide-scale electronic outreach forethought be given to management of patient expectations and flow. Before large scale implementation of such a program, validation from other geographic regions and with other age groups would be beneficial. If executed properly, electronic outreach campaigns through PHR systems may lead to increased screening, increased detection, and improved health.

## Competing interests

The authors declare that they have no competing interests.

## Authors' contributions

KM and AG wrote the initial study protocol. KM and JS conducted the focus groups. JS, BB, and KM anaylzed the data. All authors contributed to interpretation of data. KM wrote the manuscript, which was commented on by all the other authors. All authors have read and approved the final version of the manuscript.

## Supplementary Material

Additional file 1 **Focus group guides used for patients and providers**. A text file with the two patient focus group guides and the two provider focus group guides. Click here for file
