# Supplementary material for: Use of electronic personal health record systems to encourage HIV screening: an exploratory study of patient and provider perspectives
Source: BMC Res Notes. 2011 Aug 15;4:295. doi: 10.1186/1756-0500-4-295 (PMC3173346; doi:10.1186/1756-0500-4-295)
Supplement: Additional file 1 — Focus group guides used for patients and providers. A text file with the two patient focus group guides and the two provider focus group guides. [file 1756-0500-4-295-S1.DOC]

**Additional File 1. Focus group guides used for patients and providers**

**1. First Patient Focus Group – Moderator’s Guide**

Many of you have had experiences getting checked for health problems. You may have had a test for cholesterol, colon cancer, prostate cancer, or other medical conditions. We are interested in your experience with these kinds of tests, and your conversations with your doctor, nurse or physician assistant about these tests. We’re also interested in talking about tests for HIV disease. Finally we’d like to discuss whether you use the Internet and whether you use the VA website called My HealtheVet.

**Introductions:**

Please tell us your first name and something about yourself, for example something about your family, what you do for a living, or where you live.

**Screening / testing process from the patient point of view**

In today’s discussion we will be talking about your doctors, nurses, and physician assistants – and we will use the term “provider” to talk about them.

Now think back to a time when you were checked for a health problem or condition, such as having your cholesterol checked, or being tested for things like prostate or colon cancer (or any other tests you can think of).

1. Tell me how the test came about?
2. Did your provider discuss it with you? Tell me about it.
3. Has anyone here ever asked their provider for a medical test?

[PROBE: even though the provider hadn’t said you needed it.]

a. Describe the situation. What did you say to your provider?

[PROBE: How did the provider respond?]

1. **What about HIV testing? Has anyone had experience with HIV testing? What was the conversation like with your provider about HIV testing?**

**Sources of information for patients about testing**

1. Where do you get most of your information about tests and screening that might be appropriate for you?

[PROBE: doctors & nurses? Family members? TV? Books? Radio? Internet? Etc.]

1. Have you ever talked to your provider about medical tests that you read about, or heard about, or saw on television?

[PROBE: This could be written materials or just describing something that you heard or read]

1. How did your provider respond to you, and to the information?

**Patient current use of Internet & MHV for information (and transactions)**

Now I’d like to spend a few minutes discussing the Internet and a VA sponsored website.

1. How many of you use the Internet?
2. How many of you have heard of the VA website called My HealtheVet?
3. Who has looked at or registered for My HealtheVet?

For those of you who aren’t familiar with it, on the My HealtheVet website veterans can find information about the VA, learn about common health conditions, and can request medication refills. You can use My HealtheVet without registering on the website site, but if you register you can do more things on the website. There are two ways to register. One way is by entering your name and other information on the website; the other is going into a VA hospital or health center and doing it in person.

1. What have you used the My HealtheVet website for?

[PROBE: How many of you have ordered a medication refill through the My HealtheVet website?]

[PROBE: (if not clear from conversation so far): how often do you use My HealtheVet, e.g. once a week? Once a month?]

**Reactions to emails regarding HIV testing**

1. How many of you use email?

When a veteran registers for My HealtheVet he/she can provide an email address. The VA is thinking of sending health messages to veterans on the email list. One kind of email message the VA wants to send would be to encourage vets to talk to their health care provider about being tested for important diseases.

1. What do you think of this idea?

The VA is thinking of recommending that all vets get tested for HIV disease. So the first email message that the VA sends to vets might be about testing for HIV disease.

1. What do you think about this idea of an email about HIV testing?
2. What would you think or do if you got this kind of email message about HIV testing?

[PROBE: would you talk to your provider? If yes, would you set up a special appointment or wait until your next regularly scheduled appointment? ]

[PROBE: Would you call up your provider’s office to try to talk to him/her?]

1. What do you think other vets who receive the email will do?

[PROBE: call doctor’s office to ask about it? Call doctors office to set up special appointment? Wait for next regularly scheduled doctor’s visit and then ask their doctor? Ignore it (because not at risk? Why?)]

1. What do you think the email message should say?

[PROBE: What kinds of information would it need to have?]

[PROBE: Would it be good if it said –

- “Experts recommend that all adults in the US get tested for HIV”, which is what national health experts do say now]
- “Everyone should be tested…
- “The VA recommends…]

[PROBE: What do you think it shouldn’t say?]

1. The message will need to be short so that people will read it. Would it be helpful if there were a link to more information about HIV testing?
   1. What kind of information should the email link vets to?
2. Who, in the VA, should the email be from?

[PROBE: Secretary or undersecretary of the whole VA? Director of the VA facility you go to? Your primary care doctor? Someone else? Who should sign it?]

1. What should the subject line be:

[PROBE: should it say “Get an HIV test”?]

[PROBE: what kind of subject line would get your attention?]

[PROBE: What kind of subject line would make you want to delete the message before you opened it or ignore it?]

1. What else do you suggest about such an email so that you would be comfortable receiving and reading it?

**Final Questions**

I have a couple more questions, and then I have a very brief request.

1. What have we missed in our discussion?
2. Do you have any questions for me about these focus groups, and this email system to promote testing and screening?

**Suggestions for email message content**

This has been a really great discussion. I have one more request. I’d like to ask you to write down a few ideas about what the VA email message about HIV testing should say. This will be valuable information for the VA and help them in developing a good email message about HIV testing. What would be a sentence or two that you think would encourage veterans to get tested for HIV?

Please take a minute to think about this email message. Then on the paper we are handing out please write town your ideas. Please don’t put your name on the paper so that we can keep it anonymous. When you are done please fold the piece of paper and return it to us. This is of course voluntary, so if you don’t feel like writing anything just fold the piece of paper and return it to us.

===========================================================

2. **Second Patient Focus Group: Moderator’s Guide**

**Introductions:**

- **Consent form & video/recording form – discuss the purpose of the study, what is described in the consent form; provide time to read the consent form; encourage questions about the consent form; please don’t talk to others about what other focus group members have said during this focus group discussion**
- **Introduce ___________ & __________**
- **First name on card – when talking just use first name**
- **Time limitations may mean I have to interrupt a speaker**
- **Please just one person talk at a time**
- **Restrooms, snacks**
- **Ending at 3 p.m.**
- **This focus group is about ways the VA communicates with veterans about disease testing.**
- **Feel free to ask question or to ask for clarification.**
- **Also feel free to respond to what someone else in the group has said.**
- **There are no right or wrong answers. You are the experts. We are very interested in knowing about what you think about disease testing messages that are designed for veterans.**

**Please tell us your first name and something about yourself, for example something about your family, what you do for a living, or where you live.**

**In today’s discussion we will be talking about your doctors, nurses, and physician assistants – and we will use the term “provider” to talk about them.**

**CHECK THAT TAPE RECORDE IS ON.**

**Baseline Information:**

1. **How many of you have used the Internet or the Web in the past 6 months? Used email in the past 6 months? have registered for My HealtheVet? Have a cell phone?**

**[BE PREPARED TO DESCRIBE THE MY HEALTHEVET WEBSITE: A SECURE WEBSITE FOR VETERANS WHO USE THE VA HEALTH SYSTEM. IT CONTAINS INFORMATION ABOUT MANY HEALTH TOPICS. VETERANS CAN ORDER PRESCRIPTION REFILLS ON THE WEBSITE AND KEEP TRACK OF THEIR HEALTH, LIKE BLOOD PRESSURE, WEIGHT AND EXERCISE]**

**TOPIC 1:** OTHER SCREENING & HEALTH ISSUES AS CONTEXT FOR HIV SCREENING TOPIC [GOAL: FIGURE OUT IF CONCERN ABOUT HIV-RELATED HOME EMAIL IS DUE TO HIV OR DUE TO A MESSAGE COMING TO HOME EMAIL]

1. **a) How would you feel about receiving emails from the VA that give information about cholesterol and encourage you to have your cholesterol checked? [Optional: “The VA has never used email to communicate with veterans about health topics; we want to know if this is a good idea or not”]**

**b) What if the email gave you information about diabetes and encouraged you to be tested for diabetes? How would you feel about getting such an email message?**

**c) What if the email gave you information about HIV disease, and it encouraged you to be tested for HIV ? How would you feel about getting such an email message.**

**TOPIC 2:** EMAIL THAT INVITES YOU TO CLICK A LINK AND GO TO A MESSAGE AT THE MY HEALTHEVET WEBSITE. MESSAGE TOPIC IS CHOLESTEROL SCREENING.

1. **Instead of sendng an email directly to you that had information about cholesterol testing, for example, the VA could send an email with a link that you could click on that would take you to your MyHealtheVet login page. You would log in (that means entering a password that only you know), and then you would find the message from the VA. This is called a “secure” message or secure email because it is very well protected from other people seeing it. For the rest of our discussion when I say “secure message” or “secure email” I mean this kind of message that you have to use your password if you want to see it. Please look at page 1 of the handout. This is the regular email you would get from the VA. I’m also showing it on the screen. [SHOW ON POWERPOINT]**

This is an automated message from the Department of Veterans Affairs that is being sent to all veterans who have a My HealtheVet account.

There is new information for you to view about how to stay healthy. To see the information log into your “My HealtheVet” account by clicking on this web-link: https://MyHealtheVet.VA.gov.

1. **In this email, when you click on the link (the underlined part that says** [https://MyHealtheVet.VA.gov](https://MyHealtheVet.VA.gov/)**) and then enter your password, you would find the health promotion message that is on the page 2 of the handout and that I’m also showing on the screen. If you don’t use MyHealtheVet, try to imagine that you saw this secure message on your computer. [SHOW ON POWERPOINT]**

***Dear Veteran:***

***Did you know that the VA encourages veterans to get a variety of routine health tests, such as checking your cholesterol? Read below to see why.***

**Why check my cholesterol?**

Over 100 million American adults have cholesterol levels which are higher than recommended.

Having high blood cholesterol can put you at risk for heart disease, the leading cause of death in the US.

Adults aged 20 years or older should have their cholesterol checked every 5 years.

If you think you may not have had a cholesterol test in the past 5 years, ask your provider at your next visit.

***The good news!:*** Cholesterol can be lowered through diet, physical activity, weight control and medication.

1. **What do you think about the message about cholesterol testing that I have handed out and that I’m showing on the screen?**
2. **Would you discuss this message with your provider?**
3. **What would it be like talking to your provider about this message?**

**TOPIC 3:** EMAIL THAT INVITES YOU TO CLICK A LINK AND GO TO A MESSAGE AT THE MY HEALTHEVET WEBSITE. MESSAGE TOPIC IS DIABETES TESTING.

1. **What if instead of cholesterol, the secure message you received at the MyHealtheVet website was about diabetes testing? See page 3 of the handout and the message I’m showing on the screen.**

***Dear Veteran:***

***Did you know that the VA encourages veterans to get a variety of routine health tests, such as testing for diabetes? Read below to see why.***

**Why check for diabetes?**

- About 24 million Americans have diabetes, but one quarter of these people don’t know they have it, because they haven’t been tested recently.
- Diabetes can cause serious health problems like heart disease, strokes, blindness, and kidney disease.
- Adults aged 45 years or older should have their blood sugar checked (the test for diabetes) at least every 3 years.
- If you have not had your blood sugar checked in the past 3 years, or if you are unsure, ask your provider at your next visit.

***The good news!:*** If you don’t have diabetes, your provider can help you keep it that way. If you do have diabetes, your provider can tell you many ways to control it.

1. **How would you feel about this kind of diabetes communication?**
2. **Would you discuss this message with your provider?**
3. **What would it be like talking to your provider about this message?**

**TOPIC 4:** EMAIL THAT INVITES YOU TO CLICK A LINK AND GO TO A MESSAGE AT THE MY HEALTHEVET WEBSITE. MESSAGE TOPIC IS HIV TESTING.

1. **What if instead of cholesterol or diabetes, the secure message you received at the MyHealtheVet website was about HIV testing? See Page 4 of the handout and the message I’m showing on the screen.**

***Dear Veteran:***

***Did you know that the VA encourages veterans to get a variety of routine health tests, such as testing for HIV disease? Read below to see why.***

**Why check for HIV disease?**

Over 1 million Americans have HIV. Unfortunately a quarter of the people who have HIV don’t know they have it because they have never been tested.

Having HIV but not knowing you have it means that you could spread the virus to other people. Also, untreated HIV causes AIDS, which is a very serious disease.

The Centers for Disease Control (CDC) recommends that all adults get tested for HIV.

If you think you may not have been tested for HIV, or are unsure, ask your provider at your next visit.

***The good news!***: Most people tested for HIV don’t have it. But if you do have HIV you won’t lose any VA benefits, and the VA has excellent health care for HIV.

1. **How would you feel about getting this kind of HIV communication?**
2. **Would you discuss this message with your provider?**
3. **What would it be like talking to your provider about this message?**

**TOPIC 5**: BUNDLING OR PACKAGING MULTIPLE TESTING TOPICS TOGETHER TO “NORMALIZE”

1. **Another way to inform veterans about testing might be by talking about several tests at once. What would you think about getting an email that linked you to the My HealtheVet website, (like we discussed for the cholesterol testing information) and at the website you had a secure message that talked about three health issues instead of just one, specifically it talked about cholesterol, diabetes, and HIV? Please take a look at Page 5 of the Handout and the slide on the screen.**

**I’m not going to read the whole text, because it is the same text you have seen before about the three tests – cholesterol, diabetes, and HIV – but now rolled together into one message. Please take a couple of minutes to read it and think about whether it is a good idea to have the information about the three tests delivered to veterans as a single secure message.**

***Dear Veteran:***

***Did you know that the VA encourages veterans to get a variety of routine health tests, including checking your cholesterol, and testing for diabetes and HIV? Read below to see why.***

**Why check my cholesterol?**

Over 100 million American adults have cholesterol levels which are higher

than recommended.

Having high blood cholesterol can put you at risk for heart disease, the

leading cause of death in the US.

Adults aged 20 years or older should have their cholesterol checked every 5

years.

If you think you may not have had a cholesterol test in the past 5 years, ask

your provider at your next visit.

***The good news!:*** Cholesterol can be lowered through diet, physical activity, weight control and medication.

**Why check for diabetes?**

About 24 million Americans have diabetes, but one quarter of these people don’t know they have it, because they haven’t been tested recently.

Diabetes can cause serious health problems like heart disease, strokes, blindness, and kidney disease.

Adults aged 45 years or older should have their blood sugar checked (the test for diabetes) at least every 3 years.

If you have not had your blood sugar checked in the past 3 years, or if you are unsure, ask your provider at your next visit.

***The good news!:*** If you don’t have diabetes, your provider can help you keep it that way. If you do have diabetes, your provider can tell you many ways to control it.

**Why check for HIV disease?**

Over 1 million Americans have HIV. Unfortunately a quarter of the people

who have HIV don’t know they have it because they have never been tested.

Having HIV but not knowing you have it means that you could spread the

virus to other people. Also untreated HIV causes AIDS, which is a very

serious disease.

The Centers for Disease Control (CDC) recommends that all adults get

tested for HIV.

If you think you may not have been tested for HIV, or are unsure, ask your provider at your next visit.

***The good news!***: If HIV is found early, people with HIV can live long healthy lives when they take HIV medication.

**WAIT 2 OR 3 MINUTES SO PARTICIPANTS CAN READ THE TEXT**

1. **Would you discuss this message with your provider?**
2. **Would you talk to your provider about testing for all three conditions, or would you focus on just one of them? Which one?**
3. **What would it be like talking to your provider about this message?**
4. **Is it better for the VA to send one message that talks about these three tests, as shown here; or is it better for the VA to send three messages, one for each kind of test?**
5. **Why do you think one way is better than the other?**
6. **What if instead of one of these diseases we had talked about hepatitis C. How would you feel if a message encouraged you to talk to your provider about hepatitis C testing? [CAN SHOW HEP C SLIDE, IF APPROPRIATE]**
7. **How could the text of any of these messages be improved?**

-----------------------------------------------------------------------------------------------------------

**TOPIC 6**: NORMALIZING HIV DISCUSSIONS

1. **What do you think it’s like for patients to discuss HIV and HIV testing with their VA provider? Is there anything that would make it difficult? Anything that would make it easier?**
2. **Are there things the VA can do, or the local VA hospital or clinic can do, or the provider can do to make discussions of HIV and HIV testing more common between providers and patients? What kinds of things could they do?**
3. **If a provider asks a patient to be tested for HIV, what do you think might be going through the provider’s mind?**
4. **What would it be like to talk to your provider about HIV if you had seen a My HealtheVet or VA announcement recommending that everyone get tested for HIV?**

=====================================================================

**3. First Provider Focus Group – Moderator’s Guide**

## PART A: INTRO, GROUND RULES, CONSENT FORMS (10 MIN, 9-9:10)

## Introductions

- Welcome. I really appreciate you being here today and for taking the time to participate in this discussion.
- My name is __________ and I’ll be leading the discussion. I’m a health services researcher at one of the VA’s research centers of excellence, which is located at the Bedford VA; and I’m joined today by another researcher, ____________. Because we have a relatively limited amount of time, we won’t do introductions around the room, which is our typical procedure. But fortunately most of you already know each other, so that is less important here.
- This discussion is part of a number of research efforts at our center of excellence, on ways ensure that persons infected with HIV get into treatment as early as possible.
- We have invited you here today to learn about your experiences with routine screening and testing of patients, such as for high cholesterol or colorectal cancer. We’ll be asking about how you make the decision to test patients, and your experiences with a variety of tests, including HIV testing. We’ll also ask about the Internet, Email, and MyHealtheVet in connection with screening and testing. In a couple of months we hope to come back, and conduct a follow-on focus group – I’ll say a few words about that at the end.
- Today’s focus group session will run until _____ We won’t have a formal break, so please feel free to get up to use the restroom, or to help yourself to refreshments.
- Please make sure you have signed an informed consent document & an audio taping permission form.

## Ground Rules

- We are taping this session so that we can remember everything that you say. ________ and I, and other members of the study project team will listen to the tapes and use them to write up a report on this discussion. A transcription firm will write a transcript, also for use by the research team only.
- When we write our report, no one’s name will ever be used. Notice that we have only first names on the name cards. To help keep what you say confidential, please stick to firsts names. Also, to make this discussion as open and frank as possible, it is important that you agree to respect the confidentiality of each of the other members of this focus group. What individuals say in this room should be considered confidential.
- We also ask that only one person speak at a time because we don’t want to miss anyone’s comments.
- We’re interested in all of your ideas; there are no right or wrong answers here. Some of you may disagree with each other, but that’s ok: we’d actually like to hear as many different points of view as possible. Please feel free to respond to each other, and not only to me and you don’t have to wait for me to call on you to speak.
- I’ll be guiding the discussion and asking questions. Because we have limited time, I may ask you to hold your thought. Please do not take this personally. I just want to make sure we have a chance to cover all of the material.
- If you aren’t on call, please turn off your pagers and cell phones.
- Do you have any questions about the procedures or the purpose of the focus group?

**PART B: FOCUS GROUP CONTENT AREAS (35 MIN)**

**Current Systems & Procedures for Screening/Testing Patients (9:10-9:20)**

I’d like to understand the process leading to a patient getting screened or tested.

Let’s talk about routine screening, such as for things like cholesterol and for colorectal cancer, and then about HIV testing.

1. Tell me how you decide that a patient should have a diagnostic test or a screening?

PROBE: Besides clinical reminders, what else might determine whether a patient gets a test or not?

1. What about for HIV testing, how do you decide when a patient needs an HIV test?
2. Can you describe situations where patients might not get a diagnostic test they need (any kind of diagnostic test)?

PROBE: Why might patients not get a test they need?

PROBE: What about for HIV tests, why might patients not get an HIV test even though they should receive one?

PROBE: Are there situations where *the patient* is the primary reason that he or she doesn’t get an HIV test? (If patient-based reasons do not arise, i.e. patients don’t follow through or refuse). What, if any, is the patients’ role in getting or not getting tested?

1. Can you think of a time when a patient has asked you to give them a lab test? And what is that like for you?

[PROBE: Routine tests like cholesterol & colorectal cancer?]

[PROBE: And what about HIV testing?]

1. What do patients say about why they want to be tested?

[PROBE: From what information sources are they getting the idea?]

[PROBE: Can you think of a time that a patient brought in information from the Internet regarding a test they wanted you to perform?]

1. Can you think of a time when a patient came to you asking for a test that you thought was unnecessary? What happened?

[PROBE (IF HIV TESTING NOT WELL COVERED YET): Think back to a time when you ordered an HIV test for one of your patients. What were the circumstances & how did you talk about it with the patient?]

**My HealtheVet & Broadcast Email Messages about Screening (including HIV) (9:20-9:30)**

The VA has a patient website called My HealtheVet. On the website patients can find out information about the VA, learn about common health conditions, and can request medication refills.

1. How many of you have heard about My HealtheVet? What do you know about it?
2. [IF ONE OR MORE HAVE HEARD ABOUT IT: ] Have any of your patients used it, to your knowledge?
3. [IF YES] How has it affected you or your practice?

The VA is thinking of directly sending out health promotion email messages to veterans who have registered for My HealtheVet. One idea is to encourage patients to get tested and screened for various conditions (e.g. cholesterol, diabetes, hypertension, cancer). [

1. What do you think of this idea? [IF NECESSARY, “Let’s hold discussion of emails about HIV testing, and talk about less sensitive or stigmatized conditions. We’ll get to HIV shortly.”]

10a. How do you think veterans will react?

1. How might it help you in your job?
2. How might it make your job more difficult?

**HIV testing email (9:30-9:45)**

One of the first messages being proposed for emailing to Veterans might be about the importance of being tested for HIV, including the recommendation that all veterans talk to their providers about HIV testing. The VA wants patients to think of HIV testing like other routine tests, such as tests for cholesterol, prostate cancer, or colorectal cancer.

1. Based on your knowledge of your patients, how do you think they would react to this kind of email message from the VA?
2. What do you think the email message should say?
3. Who should be listed as the “sender” of the email? The VA generally? You, the clinician? others?
4. What should the subject line say?
5. How will this kind of email message to veterans affect you and your clinic?

PROBE: Your workflow? Workflow of other clinic staff, e.g. reception, nursing, social work, etc.

17a. How do you think veterans will react?

**PART C: WRAP UP & WRITING REQUEST (5 MIN)**

**Wrap Up**

I have a couple more questions and then a brief request.

1. Do you have questions for me about this focus group, and this email system to promote testing and screening? Or any last thoughts about this issue?

**Thank you very much for your ideas and your time.**

=====================================================================

**4. Second Provider Focus Group – Moderator’s Guide**

**Introductions**

- **Brief overview of the study & this focus group:**
  - **VA considering email & other electronic means to communicate with veterans about disease screening**
  - **Last fall we talked fairly broadly about this; today we have some draft email messages that we would like to share with you and get your impressions.**
  - **We’ll have you read them and then we’ll discuss them so we can learn about your impressions of the concept, as well as your thoughts on the actual content of the messages.**
- **Consent form & video/recording form – discuss the purpose of the study, what is described in the consent form; provide time to read the consent form; encourage questions about the consent form; please don’t talk to others about what other focus group members have said during this focus group discussion.**
- **Because of oversights last time we will take special care with the consent forms today. ________ will check each one and, during the discussion, she may ask you to complete something you missed.**
- **Some providers will have 2 consent forms to sign**
- **This will last an hour, not 1.5 hours as is stated on consent**
- **Introduce _____________ & ________________**
- **First name on card – when talking just use first name**
- **Time limitations may mean I have to interrupt a speaker**
- **Please just one person talk at a time**
- **Help yourself to coffee, snacks**
- **Ending at 9 a.m.**
- **This focus group is about ways the VA communicates with veterans about disease testing.**
- **Feel free to ask question or to ask for clarification during the discussion.**
- **Also feel free to respond to what someone else in the group has said.**
- **There are no right or wrong answers. You are the experts. We are very interested in knowing about what you think about disease testing messages that are designed for veterans.**
- **CHECK THAT TAPE RECORDER IS ON.**
- **Give out Handout. Please wait for us to tell you to turn to specific pages.**

**Begin Focus Group Content**

**Use of emails and MyHealtheVet website to encourage screening**

Last fall we talked to you about how the VA is considering using email or the patient website, called MyHealtheVet, to encourage patients to have various health screenings, such as for cholesterol, diabetes, and HIV. Today we’d like to review that discussion a bit, including looking at some draft messages to veterans

TOPIC 1: ELECTRONIC MESSAGES ABOUT CHOLESTEROL TESTING

Let’s talk about cholesterol screening. Imagine that the VA sent patients an email or electronic newsletter that informed them about the importance of cholesterol screening and encouraged them to talk to their VA provider about having their cholesterol checked.

Please look at page 1 of the handout. This is what the email might look like when a veteran opened it.

**Draft Email Message from VA to Veterans who Use VA health care (links to the veteran webpage, “MyHealtheVet”)**

This is an automated message from the Department of Veterans Affairs that is being sent to all veterans who have a My HealtheVet account.

There is new information for you to view about how to stay healthy. To see the information log into your “My HealtheVet” account by clicking on this web-link: https://myhealth.va.gov/

Please do not reply to this message. Messages sent to this e-mail address will NOT be reviewed or answered.

When your patient clicks on the link (the underlined part that says https://myhealth.va.gov) they would find the health promotion message that is on the second page of the handout – see page 2 of your handout.

***Dear Veteran:***

***Did you know that the VA encourages veterans to get a variety of routine health tests? Read below to see why.***

**Why check my cholesterol?**

Over 100 million American adults have cholesterol levels which are higher than recommended.

Having high blood cholesterol can put you at risk for heart disease, the leading cause of death in the US.

Adults aged 20 years or older should have their cholesterol checked every 5 years.

If you think you may not have had a cholesterol test in the past 5 years, ask your provider at your next visit.

***The good news!:*** Cholesterol can be lowered through diet, physical activity, weight control and medication.

**1.a) What would it be like for you as a provider if the VA used this kind of communication with your patients?**

**1.b) What kinds of questions do you think patients would ask you?**

**1.c) What would you talk to your patients about?**

TOPIC 2: ELECTRONIC MESSAGES ABOUT DIABETES TESTING

Now imagine that the VA sent patients an email or electronic newsletter that informed them about diabetes testing. Please look at page 3 of the handout.

***Did you know that the VA encourages veterans to get a variety of routine health tests, such as testing for diabetes? Read below to see why.***

**Why check for diabetes?**

About 24 million Americans have diabetes, but one quarter of these people don’t know they have it, because they haven’t been tested recently.

Diabetes can cause serious health problems like heart disease, strokes, blindness, and kidney disease.

Adults aged 45 years or older should have their blood sugar checked (the test for diabetes) at least every 3 years.

If you have not had your blood sugar checked in the past 3 years, or if you are unsure, ask your provider at your next visit.

***The good news!:*** If you don’t have diabetes, your provider can help you keep it that way. If you do have diabetes, your provider can tell you many ways to control it.

**2.a) What would it be like for you as a provider if the VA used this kind of communication with your patients?**

**2.b) What kinds of questions do you think patients would ask you?**

**2.c) What would you talk to your patients about?**

TOPIC 3: ELECTRONIC MESSAGES ABOUT HIV TESTING

Now imagine that the VA sent patients an email or electronic newsletter about HIV testing. Please look at page 4 of the handout.

***Dear Veteran:***

***Did you know that the VA encourages veterans to get a variety of routine health tests, such as testing for HIV disease? Read below to see why.***

**Why check for HIV disease?**

Over 1 million Americans have HIV. Unfortunately a quarter of the people who have HIV don’t know they have it because they have never been tested.

Having HIV but not knowing you have it means that you could spread the virus to other people. Also, untreated HIV causes AIDS, which is a very serious disease.

The Centers for Disease Control (CDC) recommends that all adults get tested for HIV.

If you think you may not have been tested for HIV, or are unsure, ask your provider at your next visit.

***The good news!***: Most people tested for HIV don’t have it. But if you do have HIV you won’t lose any VA benefits, and the VA has excellent health care for HIV.

**3.a) What would it be like for you as a provider if the VA used this kind of communication with your patients?**

**3.b) What kinds of questions do you think patients would ask you?**

**3.c) What would you talk to your patients about?**

TOPIC 4: ELECTRONIC MESSAGES ABOUT HIV TESTING, EMBEDDED WITH OTHER DISEASE TESTING.

Now imagine that the email or electronic newsletter talked about all three conditions – cholesterol, diabetes, and HIV – in the same message. If you look page 5 of your handout, you’ll see proposed text for such a message. Please take a couple of minutes to read the text.

***Dear Veteran:***

***Did you know that the VA encourages veterans to get a variety of routine health tests, including checking your cholesterol, and testing for diabetes and HIV? Read below to see why.***

**Why check my cholesterol?**

- Over 100 million American adults have cholesterol levels which are higher

than recommended.

- Having high blood cholesterol can put you at risk for heart disease, the

leading cause of death in the US.

- Adults aged 20 years or older should have their cholesterol checked every 5

years.

- If you think you may not have had a cholesterol test in the past 5 years, ask

your provider at your next visit.

***The good news!:*** Cholesterol can be lowered through diet, physical activity, weight control and medication.

**Why check for diabetes?**

- About 24 million Americans have diabetes, but one quarter of these people don’t know they have it, because they haven’t been tested recently.
- Diabetes can cause serious health problems like heart disease, strokes, blindness, and kidney disease.
- Adults aged 45 years or older should have their blood sugar checked (the test for diabetes) at least every 3 years.
- If you have not had your blood sugar checked in the past 3 years, or if you are unsure, ask your provider at your next visit.

***The good news!:*** If you don’t have diabetes, your provider can help you keep it that way. If you do have diabetes, your provider can tell you many ways to control it.

**Why check for HIV disease?**

- Over 1 million Americans have HIV. Unfortunately a quarter of the people

who have HIV don’t know they have it because they have never been tested.

- Having HIV but not knowing you have it means that you could spread the

virus to other people. Also untreated HIV causes AIDS, which is a very

serious disease.

- The Centers for Disease Control (CDC) recommends that all adults get

tested for HIV.

- If you think you may not have been tested for HIV, or are unsure, ask your provider at your next visit.

***The good news!***: If HIV is found early, people with HIV can live long healthy lives when they take HIV medication.

**4.a) What would it be like for you as a provider if the VA used this kind of communication with your patients?**

**4.b) What kinds of questions do you think patients would ask you?**

**4.c) What would you talk to your patients about?**

**4.d) What are the advantages of bundling the three messages together?**

**4.e) What are the disadvantages of bundling them?**

TOPIC 5: IMPROVING THE TEXT

1. **How could this electronic communication strategy that we’ve been discussing be improved?**
2. **How could the text of the messages be improved?**

TOPIC 6: SAMPLE PATIENT VIGNETTES:

I’m going to describe two vignettes about patients who received an electronic message from the VA and have come to you asking about HIV testing. Imagine they were your patients. See page 6 of the handout for the first vignette.

**The *first patient* is a 65-year old male Vietnam vet, who you are seeing for a regular check-up visit. He saw the email about the importance of HIV testing, and asks if he should be tested. He has explained to you that he has been married to the same woman for 30 years.**

**7.a) What would it be like to talk to this patient about HIV testing?**

**7.b) What would you advise him to do?**

**Now turn to page 7 for the second patient vignette.**

**The *second patient* is a 22-year old male vet of the Iraq war who you are seeing for a regular check-up visit. He doesn’t think he needs to be tested because he is in a monogamous relationship; he’s just asking because he saw the email that said all vets should talk to their provider about HIV testing.**

**8.a) What would it be like to talk to this patient about HIV testing?**

**8.b) What would you advise him to do?**
